# MSC Exosomes and Rutin‐Chitosan–Pectin Nanoparticles Synergize to Ameliorate Adjuvant Arthritis via Th1/Th2 Modulation, MMP Suppression, Nrf2 Upregulation, and Antioxidant Boost

**DOI:** 10.1155/sci/3586025

**Published:** 2026-04-03

**Authors:** Karim M. Moftah, Walaa G. Hozayen, Nabil A. Hasona, Hessah M. Al-Muzafar, Kamal A. Amin, Hussah A. Alshwyeh, Khairy M. A. Zoheir, Osama M. Ahmed

**Affiliations:** ^1^ Department of Biochemistry, Faculty of Science, Beni-Suef University, Beni-Suef, Egypt, bsu.edu.eg; ^2^ Department of Chemistry, College of Science, Imam Abdulrahman Bin Faisal University, Dammam, Saudi Arabia, iau.edu.sa; ^3^ Basic and Applied Scientific Research Center (BASRC), Imam Abdulrahman Bin Faisal University, Dammam, Saudi Arabia, iau.edu.sa; ^4^ Department of Biology, College of Science, Imam Abdulrahman Bin Faisal University, Dammam, Saudi Arabia, iau.edu.sa; ^5^ Department of Cell Biology, Biotechnology Research Institute, National Research Centre, Cairo, Egypt, nrc.sci.eg; ^6^ Physiology Division, Department of Zoology, Faculty of Science, Beni-Suef University, Beni-Suef, Egypt, bsu.edu.eg

**Keywords:** antioxidant, BM-MSCs, exosomes, oxidative stress, rutin, rutin-chitosan-pectin nanoparticles

## Abstract

**Background:**

Due to toxicity, high costs, and potential side effects of standard treatments of rheumatoid arthritis (RA) including nonsteroidal anti‐inflammatory drugs (NSAIDs), corticosteroids, and disease‐modifying antirheumatic drugs (DMARDs), natural products and advanced drug delivery systems, such as nanoparticles and mesenchymal stem cell (MSC)–derived exosomes (EXO), have garnered interest due to their ability to target inflammation and oxidative damage, with enhanced precision and reduced side effects, offering a promising approach for RA management.

**Methods:**

EXO were isolated from the conditioned medium of bone marrow–derived MSCs (BM‐MSCs). Rutin (RT)‐loaded chitosan (Cs)/pectin nanoparticles were prepared using a modified ionic gelation technique to enhance stability and bioavailability. Sixty male Wistar rats were utilized in the in vivo experiment and randomly assigned to six groups, each comprising 10 animals. These groups were (1) normal control, (2) complete Freund’s adjuvant (CFA)‐induced arthritic control, (3) CFA‐induced arthritis treated with free RT (20 mg/kg), (4) CFA‐induced arthritis treated with EXO (100 µg protein per rat, intravenous injection, once weekly), (5) CFA‐induced arthritis treated with RT‐Cs–pectin nanocomposite (RT‐CPN) (20 mg/kg), and (6) CFA‐induced arthritis treated with a combination of RT‐CPN and EXO. Treatments were administered for 28 days, after which the rats were euthanized for further analysis. For molecular evaluations, blood was collected for serum isolation, and the right ankle joint was carefully dissected.

**Results:**

Treatment with RT, EXO, RT‐CPN, and especially, EXO + RT‐CPN combination significantly reduced serum levels of anticitrullinated protein antibodies (ACPAs), interleukin‐1β (IL‐1β), interleukin‐6 (IL‐6), and the marker of oxidative stress malondialdehyde (MDA). These treatments also decreased inducible nitric oxide synthase (iNOS) mRNA expression, a key regulator of oxidative and inflammatory processes. Conversely, antioxidant defenses improved, as indicated by increased serum glutathione (GSH), interleukin‐10 (IL‐10), and interleukin‐13 (IL‐13) levels, along with upregulation of antioxidant enzymes such glutathione peroxidase (GPx), glutathione S‐transferase (GST), glutathione reductase (GR), and superoxide dismutase (SOD). Joint degradation was notably reduced by suppressing the protein levels of MMP‐1, MMP‐3, MMP‐9, and MMP‐13, while nuclear factor erythroid 2–related factor 2 (Nrf2) expression, a critical regulator of cellular protection, was elevated. Along with improvements in functional and molecular markers, the right hind leg’s swelling and redness decreased, and the histological alterations including pannus development, inflammatory cell infiltrations, synovial membrane hyperplasia, and degradation of articular cartilage were substantially suppressed after treatments.

**Conclusions:**

The combination of EXO + RT‐CPN demonstrated the strongest antiarthritic effects, reducing inflammation, oxidative stress, and joint degradation while boosting the body’s antioxidant defenses. These findings highlight a promising, safer therapeutic strategy for RA management.

## 1. Introduction

Rheumatoid arthritis (RA) is an inflammatory disorder marked by advancing synovial membrane swelling, joint damage, and functional impairment [1]. RA is distinguished by synovial inflammation, which involves pannus formation, histiocyte proliferation, infiltration of inflammatory cells, elevated expression of cell surface adhesion molecules, and altered protease and cytokine levels [2]. This inflammatory environment is largely driven by proinflammatory cytokines like tumor necrosis factor (TNF‐α), interleukin‐1β (IL‐1β), and interleukin‐6 (IL‐6), which trigger NF‐κB signaling. As a result, oxidative stress rises, and macrophages shift toward a proinflammatory state, perpetuating synovitis and ongoing tissue damage [3, 4]. RA impacts over 20 million people each year, mainly middle‐aged adults, with a 0.5%–1% prevalence in industrialized nations. Women are at a risk that is 2.5–3 times higher than that of men [5]. Genetic factors, environmental influences, and immune dysfunction all contribute to the development of RA, leading to the uncontrolled release of proinflammatory cytokines and the activation of matrix metalloproteinases (MMPs) [6]. Zinc‐dependent MMPs degrade the extracellular matrix, contributing to cartilage deterioration and joint deformities [7]. Reactive oxygen species (ROS) increase inflammation, and tissue damage contributes to RA development [8]. The primary treatment for RA involves medications, which are typically categorized into three groups: disease modifying antirheumatic drugs, nonsteroidal anti‐inflammatory drugs (NSAIDs), and biological agents. However, these medications often entail noticeable side effects. As a result, scientists are increasingly focused on discovering natural‐derived antiarthritic therapies that are both safer and more efficacious [9, 10]. Rutin (RT) (3,3,4,5,7‐pentahydroxyflavone‐3‐rhamnoglucoside) is a naturally occurring flavonoid found in various plants, including apples, buckwheat, tea, and passion flowers. It exhibits diverse therapeutic properties, such as antimicrobial, antifungal, vasoprotective, anti‐inflammatory, antioxidant, neuroprotective, and cardioprotective activities [11].

Importantly, RT has shown antiarthritic potential in experimental models by attenuating paw swelling, suppressing proinflammatory mediators, and restoring antioxidant defenses [12, 13]. However, RT’s therapeutic potential is limited by its poor stability and low bioavailability, which are primarily attributed to its low solubility in water [14]. By using specialized nanocarriers and reducing particle size, nanotechnology largely increases the solubility and dissolution rate of weakly water‐soluble medications, greatly improving their stability and bioavailability [15–17]. Nanoparticles have been created as a successful method to address these issues by boosting RT’s solubility, stability, and targeted administration, eventually increasing its therapeutic potential [13]. In this regard, biopolymer carriers such as chitosan (Cs) and pectin are highly favored because they are biocompatible and biodegradable while also enhancing drug encapsulation, improving mucosal adhesion, increasing cellular uptake, and allowing for sustained release of poorly soluble compounds [13, 18–20].

Exosomes (EXO) are extracellular vesicles (30–150 nm) released by various cells, such as mesenchymal stem cells (MSCs), immune cells, and synovial fibroblasts. They facilitate cell communication by carrying proteins, lipids, and microRNAs, impacting immune regulation, inflammation, and tissue repair. Their immunomodulatory properties make them promising therapeutic agents for autoimmune diseases like RA [21]. Recent studies show that MSC‐derived EXO help decrease synovial inflammation by modulating T cell responses and encouraging the polarization of macrophages toward an anti‐inflammatory state while also aiding in joint regeneration, positioning them as a promising cell‐free treatment for RA [22]. However, the potential synergistic antiarthritic effects of combining a bioavailability‐enhanced RT nanoformulation with immunomodulatory EXO remain insufficiently investigated.

Therefore, the aim of this study was to evaluate the therapeutic efficacy of RT‐Cs–pectin nanoparticles (RT‐CPN), EXO, and their combination in complete Freund’s adjuvant (CFA)–induced arthritis, with particular emphasis on anti‐inflammatory, antioxidant, and immunomodulatory mechanisms.

## 2. Materials and Methods

### 2.1. Experimental Animals

From December 2022 to May 2023, 60 male Wistar rats weighed 120–140 g and aged 7 to 8 weeks were subjected to study at the Faculty of Science at Beni‐Suef University, Egypt. The rats were obtained from the animal house in Helwan and Egyptian Organization for Biological Products and Vaccines (VACSERA). The animals were closely monitored for ~8 days before starting the study to ensure that there were no concurrent infections. In ventilated polypropylene cages covered with stainless steel, standard, balanced food and water at room temperature (between 25 ± 5°C) were freely available to the animals. Animal weight was recorded weekly throughout the experiment. Under the ethical approval number 022/331, the ethical guidelines of Beni‐Suef University’s Institutional Animal Care and Use Committee (IACUC), Egypt, were strictly followed during animal handling and experimental procedures. We made every possible effort to minimize both the animal population involved and the extent of their distress and pain.

### 2.2. Chemicals

Santa Cruz Biotechnology, Inc. (10410 Finnell Street Dallas, TX 75220, USA), supplied Freund’s complete adjuvant (FCA), a suspension of heat‐inactivated *Mycobacterium tuberculosis* in mineral oil (1 mg/mL). Oxford Laboratory (Mumbai, India) provided the RT or vitamin P. Cs and sodium tripolyphosphate (TPP) were acquired from Sigma–Aldrich Chemical Co. (St. Louis, MO, USA). Merck, a German firm from Darmstadt, supplied the pectin. Every other reagent and solvent that was employed was of analytical quality.

### 2.3. MSC–Conditioned Media (MSC‐CM)

To obtain MSC‐conditioned medium (MSCs‐CM), MSCs were grown in Dulbecco’s modified eagle medium (DMEM) and allocated into 50 mL Falcon tubes containing 45 mL of medium within a laminar airflow environment. These tubes were maintained at 2–8°C. An antibiotic vial, stored at −20°C, was thawed in a water bath for 20 min, aliquoted into 10–15 mL portions, and transferred to 15 mL tubes. The serum‐free medium was prepared by combining 45 mL of DMEM with 0.5 mL of the thawed antibiotic solution. MSCs were seeded at a density of 10,000 cells/cm^2^ and cultured in complete media for 24 h. Following three washes with phosphate‐buffered saline (PBS), they were incubated in serum‐free medium for 48 h. The supernatants were then collected, filtered, and concentrated by centrifugation at 12,000 rpm [23, 24].

#### 2.3.1. MSC Characterization

Cultured MSCs were expanded under standard sterile conditions in appropriate growth medium until they reached ~80%–90% confluence. Cells were harvested by enzymatic dissociation, washed, and incubated with fluorophore‑conjugated antibodies against surface markers including CD105 and CD73 as positive markers and CD45 as a negative marker. Flow cytometry (BD flow cytometer) was performed for MSC immunophenotyping to assess purity and identity.

### 2.4. Isolation and Characterization of EXO

MSC‐CM were initially centrifuged at 300 g for 30 min at 4°C to remove entire cells and bigger debris. To separate the microvesicle (MV) fraction, the supernatant was centrifuged at 16,500 g for 20 min at 4°C using a Sorvall MTX150 (Thermo Scientific). The pellet was washed with PBS, centrifuged at 16,500 g for 20 min at 4°C, then resuspended in PBS, and preserved at −80°C. To extract the EXO fraction, the remaining supernatant was centrifuged at 120,000 g for 2.5 h at 4°C. The EXO pellet was stored at −80°C after being reconstituted in PBS [25, 26].

EXO were characterized using high‐resolution transmission electron microscopy (HR‐TEM) (JEOL JEM‐2100, Tokyo, Japan) to visualize their morphology and determine their size. Furthermore, flow cytometry was performed to characterize EXO based on established exosomal markers. Due to the small size of EXO, vesicles were first adsorbed onto aldehyde/sulfate latex beads (4 µm diameter). The bead‐bound EXO were subsequently stained with Alexa Flour 488‐conjugated antibodies specific for HSP70, CD9, CD63, and CD81 (Elabscience, China). Bead‐bound EXO were blocked with 1% bovine serum albumin (BSA) in PBS to prevent nonspecific binding. After staining and washing, samples were analyzed on a BD flow cytometer, and data were processed using FlowJo software.

#### 2.4.1. EXO Size and Size Distribution Analysis by Dynamic Light Scattering (DLS)

The hydrodynamic diameter of the isolated EXO was determined using a Zetasizer instrument (Malvern ZetaSizer, UK) based on DLS. EXO samples were diluted in sterile, filtered PBS to obtain an appropriate concentration and to avoid multiple scattering effects. Measurements were performed at 25°C.

### 2.5. Preparation of RT‐Loaded Cs‐Pectin (Core‐Shell) Nanoparticles (RT‐CPN)

A modified ionic gelation method was employed to formulate RT‐loaded Cs nanoparticles (Cs NPs) [27]. Initially, 0.3 g of Cs was dissolved in 100 mL of distilled water and then added 1% glacial acetic acid (v/v). Using 1 M NaOH, the solution PH was adjusted to six with continuous magnetic stirring at 1000 r.p.m. for 1 h. Then, 100 mg of powdered RT, dissolved in 4 mL of the DMSO solution, was gradually added to the Cs solution, and the mixture was kept at 1000 r.p.m. with magnetic stirring for 1 h. Subsequently, 0.1 g of TPP was dissolved in distilled water and added slowly drop by drop to the mixture at constant stirring for 1 h at 1500 r.p.m. RT‐loaded Cs NPs were obtained by sonicating the resultant mixture for 1 h. Additionally, blank Cs NPs were prepared using a similar method but without adding RT Drug.

First, 0.5 g of powdered pectin was dissolved in distilled water to create the pectin solution. For 30 min, the mixture was heated to 70°C while being constantly stirred. The solution was then let to cool to ambient temperature while being agitated for another hour. 0.1 M HCl was added to the solution dropwise to get the required pH of 6.0. The resultant mixture was maintained at 1000 r.p.m. with magnetic stirring.

A previously published protocol was modified to synthesize RT‐CPNs [28]. RT‐loaded Cs NPs were mixed with 20 mL of pectin solution in water and stirred continuously for 30 min. The resulting suspension was centrifuged at 6000 rpm for 20 min. The settled nanoparticles were then freeze‐dried for 48 h to yield RT‐CPNs.

### 2.6. Initiation of Adjuvant‐Induced Arthritis

To induce arthritis, the plantar regions of the rat’s right hind paw were subcutaneously injected with 100 µL CFA/rat/day for two successive days [9].

### 2.7. Animal Grouping

**Figure 1:**
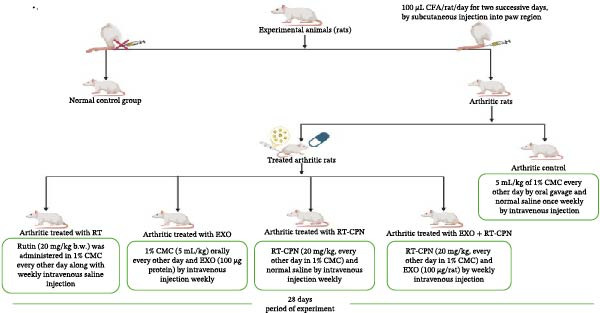
Animal grouping and design of the experiment.

After being injected with CFA to develop RA, Wistar rats were separated into six groups of 10, as indicated in Figure 1.

Group 1 (normal control group): For 28 days, rats in this group were administered an oral dose of the vehicle (1% carboxymethyl cellulose [CMC]) equal to 5 mL/kg/every other day and an intravenous injection of normal saline once a week.
Group 2 (arthritic control group): Arthritic rats in this group received orally the same amount of vehicle (1% CMC, 5 mL/kg/every other day) and were intravenously injected with a normal saline once a week as rats in the normal control group for 28 days.
Group 3 (arthritic treated with RT): For 28 days, arthritic rats received RT orally at 20 mg/kg every other day [29] dissolved in 5 mL of 1% CMC, along with weekly intravenous injections of saline.
Group 4 (arthritic treated with EXO): For 28 days, arthritic rats in this group were intravenously injected with EXO once a week (100 µg protein per rat) [30] and was given an equal volume of the vehicle (1% CMC, 5 mL/kg, administered every other day).
Group 5 (arthritic treated with RT‐CPN): For 28 days, arthritic rats in this group had received an optimized dose of RT‐CPN (20 mg/kg/ every other day dissolved in 5 mL of 1% CMC) and intravenous injection of normal saline once a week.
Group 6 (arthritic treated with EXO + RT‐CPN mixture): For 28 days, arthritic rats in this group received both an optimized dose of RT‐CPN (20 mg/kg/ every other day dissolved in 5 mL of 1% CMC) and intravenous injection of EXO once a week (100 µg protein per rat).

Upon completion of the study period, rats were anesthetized via inhalation of diethyl ether. Blood samples from the jugular vein were placed in coagulation tubes and centrifuged for 15 min at 3000 rpm. Sera were divided into three aliquots per animal and stored for subsequent analysis at −20°C. Three rats from each group had their right rear ankles excised and stored in 10% NBF for histological analysis. Furthermore, three rats from each group had their right hind ankles preserved at −80°C for Western blot analysis.

### 2.8. Histopathological Investigation

Following the sacrifice and dissection of the animals, the right ankle joints were properly removed and preserved in 10% neutral buffered formalin (NBF) for 48 h. Upon completion of fixation, the samples were processed for histopathological examination. The ankle joints were decalcified in a 10% hydrochloric acid solution to facilitate appropriate sectioning. The decalcification process was observed over a fortnight by delicately probing the tissue with a surgical blade, and the acid solution was replenished biweekly to preserve its efficacy. Following thorough decalcification, the samples were further dehydrated using a sequence of ethanol concentrations and subsequently embedded in paraffin wax. Thin sections, 5 µm in thickness, were prepared and stained with hematoxylin and eosin according to Bancroft and Gamble [31].

### 2.9. Quantification of Biomarkers Using Enzyme‐Linked Immunosorbent Assay (ELISA)

Serum concentrations of anticitrullinated protein antibodies (ACPAs), IL‐1β, IL‐6, interleukin‐10 (IL‐10), and interleukin‐13 (IL‐13) were assessed using specific ELISA kits, following the protocols established by the manufacturer. ELISA kits from Wuhan Fine Biotech Co., Ltd. (Wuhan, Hubei, China) were utilized to quantify ACPA levels. An ELISA kit from CUSABIO (Houston, TX, USA) was used to quantify IL‐13, while ELISA kits from Cloud‐Clone Corp. (23603W. Fernhurst Dr., Unit 2201, Katy, TX 77494, USA) were employed to identify IL‐1β, IL‐6, and IL‐10. Six samples were detected in each group for each parameter, and all detections were performed according to manufacturers’ instructions. Concentrations were calculated using standard curves and are expressed in pg/mL for IL‐1β, IL‐6, IL‐10, and IL‐13 and ng/mL for ACPA. Absorbance readings were taken at 450 nm using a microplate reader to determine biomarker levels.

### 2.10. Colorimetric Assay

The concentrations of reduced glutathione (GSH) and malondialdehyde (MDA) were determined using BioVision’s colorimetric assay kits (Milpitas, CA, USA) according to the manufacturer’s guidelines. For GSH quantification, serum was incubated with provided reagents, and absorbance was measured at 450 nm. For MDA, serum samples were reacted with thiobarbituric acid (TBA) to form an MDA‐TBA adduct, which was quantified by measuring absorbance at 532 nm. Concentrations were calculated from standard curves. The results were expressed as nmol/mg for GSH and MDA.

### 2.11. Analysis of Protein Expression *via* Western Blot

Western blot analysis was conducted to evaluate the expression of MMPs (MMP‐1, MMP‐3, MMP‐9, and MMP‐13) and nuclear factor erythroid 2‐related factor 2 (Nrf2) in rat ankle tissue samples. Protein extraction was performed using the ReadyPrep Protein Isolation Kit (Bio‐Rad Inc., Hercules, CA, USA), and protein concentrations were determined with the Bradford assay (Bio Basic Inc., Markham, Ontario, Canada). Equal amounts of protein (20 μg) were mixed with Laemmli sample buffer and denatured by boiling at 95°C for 5 min. Proteins were separated by Sodium Dodecyl Sulfate–Polyacrylamide Gel Electrophoresis (SDS‐PAGE) using the TGX Stain‐Free FastCast Acrylamide Kit (Bio‐Rad Laboratories, Inc., Hercules, CA, USA). The proteins were transferred to a PVDF membrane using the Bio‐Rad Trans‐Blot Turbo system. The membrane was then blocked for 1 h at room temperature in Tris‐buffered saline with Tween 20 (TBST) containing 3% BSA. The membranes were then incubated overnight at 4°C with primary antibodies specific to MMP‐9 (Cell Signaling Technology, Danvers, MA, USA), MMP‐1, MMP‐3, and Nrf2 (Santa Cruz Biotechnology, Dallas, TX, USA), as well as MMP‐13 (Proteintech Group, Rosemont, IL, USA). After washing with TBST, membranes were treated with horseradish peroxidase (HRP)–conjugated secondary antibodies (Novus Biologicals) for 1 h at room temperature. Protein bands were visualized using Clarity Western ECL Substrate (Bio‐Rad Laboratories Inc., Hercules, CA, USA), and detection was performed using a CCD camera–based imaging system. Band intensities were quantified and normalized to β‐actin to account for loading variations.

### 2.12. Reverse Transcription‐Polymerase Chain Reaction (RT‐PCR) Analysis

The expression levels of mRNA for inducible nitric oxide synthase (iNOS), glutathione peroxidase (GPx), glutathione S‐transferase (GST), glutathione reductase (GR), and superoxide dismutase (SOD) were determined by employing RT‐PCR. Total RNA was isolated with the Direct‐zol RNA Miniprep Plus Kit (Zymo Research, USA). RNA purity, integrity, and concentration were subsequently quantified using a Beckman Coulter dual‐beam spectrophotometer calibrated at 260 nm. Reverse transcription and subsequent PCR amplification were conducted with the SuperScript IV One‐Step RT‐PCR Kit (Thermo Fisher Scientific, USA). RT‐PCR was performed using gene‐specific primers for iNOS, SOD, GPx, GST, and GR, with GAPDH as the housekeeping gene. The reaction mix, consisting of 25 µL of 2x Platinum SuperFi RT‐PCR Master Mix, 0.5 µL of SuperScript IV RT Mix, 2.5 µL of each primer (10 µM), 10 µL RNA template, and 9.5 µL nuclease‐free water, was amplified on the Step One Real‐Time PCR System (Applied Biosystems, USA). Thermal cycling conditions included reverse transcription at 55°C for 10 min, enzyme inactivation at 95°C for 2 min, followed by 40 cycles of denaturation at 95°C for 10 s, annealing at 55°C for 10 s, and extension at 72°C for 30 s, with a final extension at 72°C for 5 min. Relative expression levels were calculated using the ΔΔCt technique (2^−ΔΔCt^). The primer sequences utilized for amplification are listed in Table 1.

**Table 1:** Forward and reverse primer sequences of targeted genes.

Gene	Forward sequence	Reverse sequence	Gene accession number
iNOS	TTC TTT GCT TCT GTG CTA ATG CG	GTT GTT GCT GAA CTT CCA ATC GT	NM_012611.3
*SOD*	TTCGAGCAGAAGGCAAGCGGTGAA	AATCCCAATCACACCACAAGCCAA	NM011434
*GSH*	TACGGCTCACCCAATGCTC	CTATGGCACGCTGGTCAAATA	XM_039104373.1
*GPX*	GTGCGAAGTGAATGGTGAGA	CTGGGACAGCAGGGTTTCTA	NM008160
*GST*	TAGACAGTGAAGCTTAGTTGCTGCT	CAAGAAATTGGACACTGCAGCTCC	XM_032909713.1
*GAPDH*	CAATGACCCCTTCATTGACC	GACAAGCTTCCCGTTCTCAG	DQ40057.1

### 2.13. Statistical Analysis

Statistical analyses were conducted using SPSS software (version 25, Chicago, IL, USA). Differences among group means were assessed via one‐way analysis of variance (ANOVA), with Tukey’s post hoc test applied for multiple comparisons. Results are expressed as mean ± standard error of the mean (SE). The number of detected samples in each group for the parameters detected by ELISA and colorimetric analysis was six, while the number for parameters detected by Western blot and RT‐PCR analysis was three replicates for each group. Statistical significance was defined as a *p*‐value less than 0.05 (*p*  < 0.05).

## 3. Results

### 3.1. Effect of Treatments on Gross Lesions of the Paw and Ankle Joint

The severity of arthritic inflammation was assessed by observing edema and erythema in the right hind paw as external indicators. In the CFA‐induced arthritic control group, these parameters progressively diminished subsequent to treatments with RT, EXO, RT‐CPN, and the EXO + RCPN mixture (Figure 2).

Figure 2The physical condition of the right hind leg paw of rats reveals edema and inflammation among different groups of rats. (A) Normal rat. (B) Arthritic control rat. (C) Arthritic rat treated with RT. (D) Arthritic rat treated with EXO. (E) Arthritic rat treated with RT‐CPN. (F) Arthritic rat treated with EXO + RCPN mixture.

**Figure:**
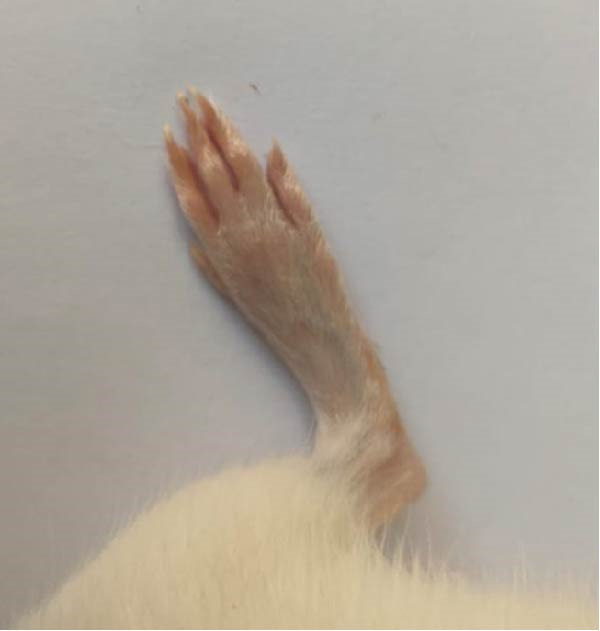
(A)

**Figure:**
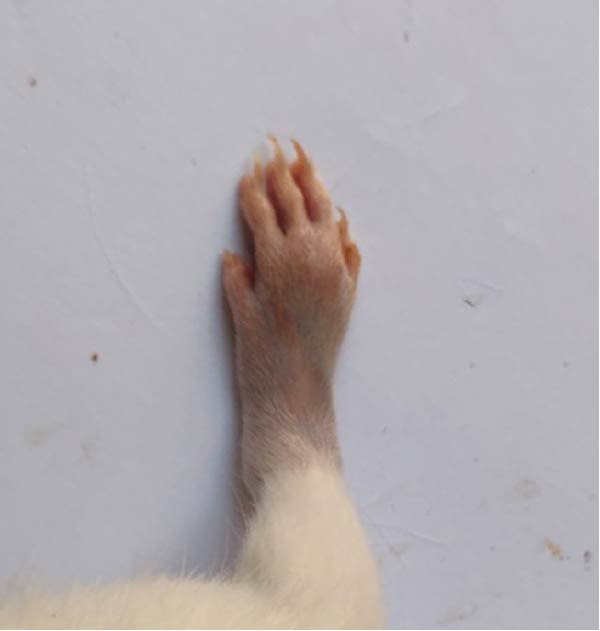
(B)

**Figure:**
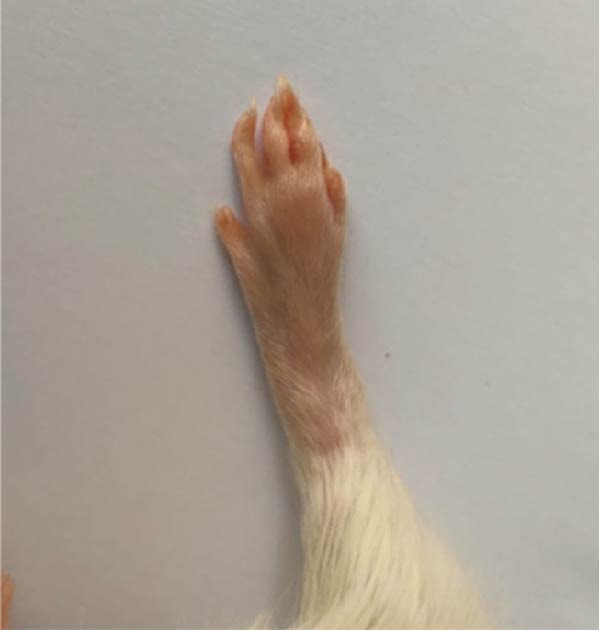
(C)

**Figure:**
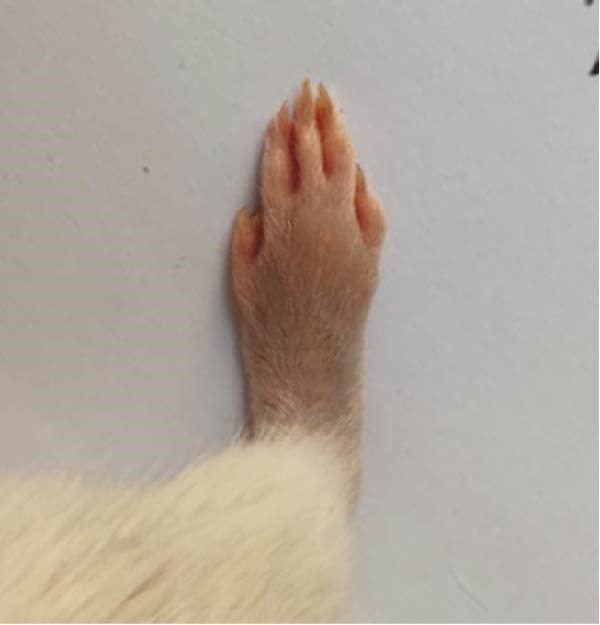
(D)

**Figure:**
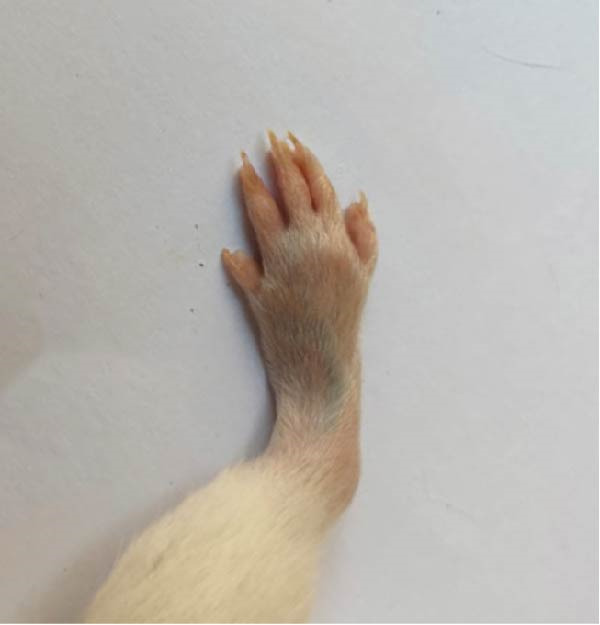
(E)

**Figure:**
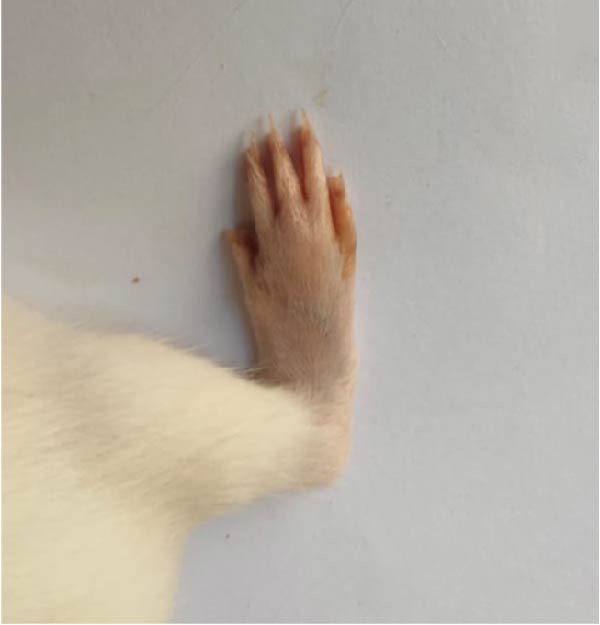
(F)

### 3.2. Flow Cytometric Characterization of MSCs

**Figure 3:**
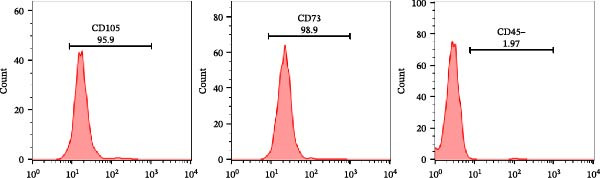
Flow cytometric analysis for MSC markers.

Flow cytometric analysis of the cultured MSC population demonstrated a phenotype consistent with established MSC criteria. The majority of cells expressed the characteristic mesenchymal markers, with 95.9% positive for CD105 and 98.9% positive for CD73, while the expression of the hematopoietic marker CD45 remained low at 1.97% as shown in Figure 3. These results confirm that the culture predominantly comprised MSCs with minimal contamination from nonmesenchymal cells, aligning with reported marker profiles for human MSCs in the literature.

### 3.3. Transmission Electron Microscopy (TEM) Photomicrographs of BM‐MSC–Derived EXO and RT‐CP Nanoparticles

**Figure 4:**
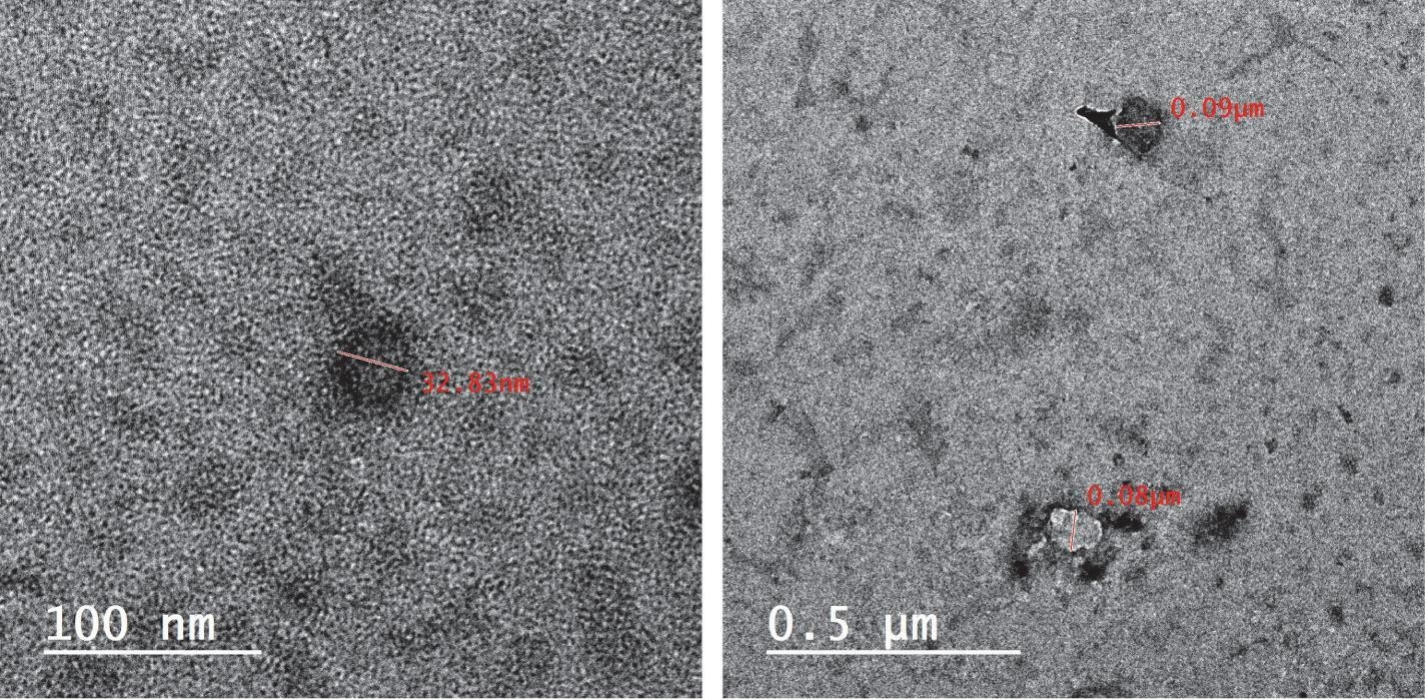
The images of BMSCS‐EXO by TEM.

**Figure 5:**
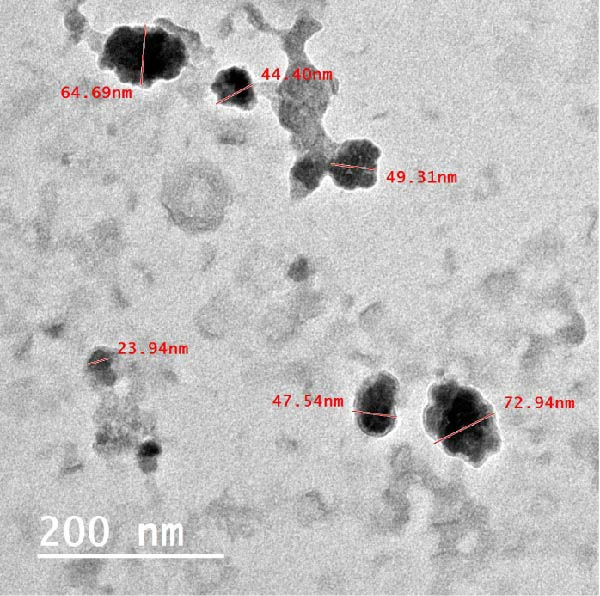
TEM of RT‐CPN.

TEM showed that the EXO were between 33 and 90 nm in size and had the typical cup form with a double membrane around them (Figure 4). TEM investigation showed that the RT‐CPN particles were spread out evenly and not stuck together, with sizes ranging from 23 to 73 nm, and all full characterizations of RT‐CPN were mentioned in our previous publication [32] (Figure 5).

### 3.4. Flow Cytometric Characterization of EXO

**Figure 6:**
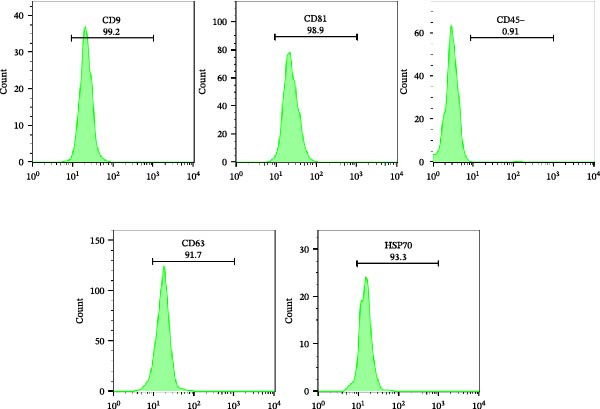
Flow cytometric analysis for exosome markers.

Phenotypic profiling of isolated extracellular vesicles indicated high positivity for canonical EXO markers. Specifically, 99.2% of vesicles were positive for CD9 and 98.9% for CD81, with 91.7% positive for CD63. Additionally, the intracellular protein HSP70 was detected in 93.3% of EVs, supporting the presence of EXO‑associated proteins. In contrast, only 0.91% of vesicles exhibited CD45 expression as shown in Figure 6, indicating negligible contamination from residual cellular debris or non‑EV particles. The combination of high positivity for tetraspanins and HSP70 with minimal CD45 expression is consistent with established criteria for MSC‑derived EXO characterization.

### 3.5. Size Distribution and Homogeneity of EXO

**Figure 7:**
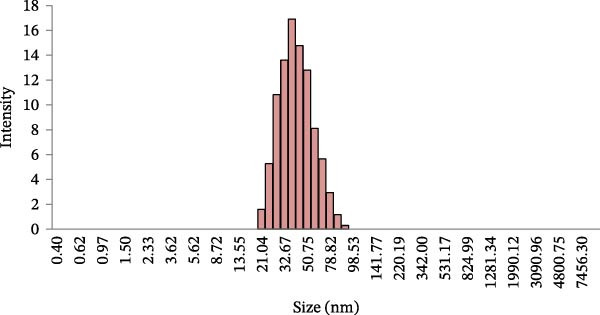
BMSCS‐EXO size distribution.

DLS analysis revealed that the isolated EXO had a mean hydrodynamic diameter of 38.62 nm. In addition, the polydispersity index (PDI) was 0.230, indicating a relatively narrow size distribution and a homogeneous population of vesicles (Figure 7).

A PDI value below 0.3 suggests minimal aggregation and good sample uniformity. These findings confirm that the isolated vesicles fall within the expected nanoscale range for EXO and demonstrate the reliability of the isolation method. When combined with flow cytometric detection of exosomal markers, the data further validate the successful characterization of EXO.

### 3.6. Effects on Serum ACPA and Different ILs

The effects of RT, EXO, RT‐CPN, and EXO + RT‐CPN combination on serum levels of IL‐1β, ACPA, IL‐6, IL‐10, and IL‐13 in arthritic rats are shown in Table 2. Relative to the normal control group, arthritic rats showed marked increases in ACPA, IL‐1β, and IL‐6 levels by 266%, 810.42%, and 469.80%, respectively, while IL‐10 and IL‐13 levels declined by 64.46% and 57.63%. Administration of RT, EXO, RT‐CPN, and their combination (EXO + RT‐CPN) significantly lowered the elevated ACPA, IL‐1β, and IL‐6 levels and restored IL‐10 and IL‐13 levels. Of all treatments, the combined EXO +RT‐CPN therapy produced the most substantial changes in serum markers, with percentage changes of −61.939% for ACPA, −79.196% for IL‐1β, −73.982% for IL‐6, 201.675% for IL‐10, and 137.762% for IL‐13.

**Table 2:** Effects of RT, EXO, RT‐CPN, and EXO + RT‐CPN on serum levels of ACPA, IL‐1β, IL‐6, IL‐10, and IL‐13 in CFA‐induced arthritic rats.

Groups	Parameters				
	ACPA (ng/mL)	IL‐1β (pg/mL)	IL‐6 (pg/mL)	IL‐10 (pg/mL)	IL‐13 (pg/mL)
Normal control	8.733 ± 0.928^a^	20.767 ± 2.132^a^	18.100 ± 1.321^a^	50.267 ± 4.922^c^	203.767 ± 7.660^d^
Arthritic control	31.967 ± 1.224^d^	189.426 ± 8.426^d^	103.133 ± 7.158^d^	17.867 ± 1.464^a^	86.333 ± 2.066^a^
Arthritic treated with RT	16.200 ± 0.842^b,c^	95.333 ± 3.579^c^	45.900 ± 1.805^c^	36.100 ± 2.482^b^	136.967 ± 5.196^b^
Arthritic treated with EXO	15.033 ± 0.697^b,c^	68.233 ± 3.264^b^	37.000 ± 1.177^b,c^	46.700 ± 3.551^b,c^	181.600 ± 4.805^c^
Arthritic treated with RT‐CPN	18.333 ± 1.131^c^	79.633 ± 3.723^b,c^	50.600 ± 3.768^c^	40.833 ± 2.671^b,c^	147.600 ± 4.134^b^
Arthritic treated with EXO + RCPN	12.167 ± 0.664^a,b^	39.333 ± 2.953^a^	26.833 ± 1.853^a,b^	53.900 ± 2.744^c^	205.267 ± 4.087^d^

*Note*: Data are presented as mean ± standard error, with six samples detected per group. Matching superscript symbols within a column denote no statistically significant difference.

### 3.7. Effects on Lipid Peroxidation Products and Cellular Antioxidant Defense Mechanisms

In the arthritic group, serum MDA levels increased by 334.93%, while serum GSH levels decreased by 74.25% compared to the normal control group. Treatment with RT, EXO, RT‐CPN, or the combination of EXO +RT‐CPN resulted in significant reductions in serum MDA levels by 40.02%, 63.79%, 46.51%, and 63.05%, respectively. Additionally, these treatments elevated serum GSH levels by 92.36%, 192.04%, 111.47%, and 215.37%, respectively, compared to untreated arthritic rats, as shown in Table 3.

**Table 3:** Effect of RT, EXO, RT‐CPN, and the combination between EXO and RT‐CPN on serum GSH and MDA levels.

Groups	Parameters	
	GSH (nmol/mg protein)	MDA (nmol/mg protein)
Normal control	1.693 ± 0.059^d^	0.373 ± 0.038^a^
Arthritic control	0.436 ± 0.081^a^	1.621 ± 0.059^d^
Arthritic treated with RT	0.839 ± 0.042^b^	0.972 ± 0.058^c^
Arthritic treated with EXO	1.273 ± 0.052^c^	0.587 ± 0.064^b^
Arthritic treated with RT‐CPN	0.922 ± 0.043^b^	0.867 ± 0.027^c^
Arthritic treated with EXO + RCPN	1.375 ± 0.069^c^	0.599 ± 0.057^b^

*Note*: Data are presented as mean ± standard error, with six samples detected per group. Matching superscript symbols within a column denote no statistically significant difference.

In arthritic rats, the activity of antioxidant enzymes GR, SOD, GPx, and GST in the ankle was markedly reduced by 85.644%, 94.270%, 82.474%, and 77.481%, respectively, compared to the normal group. Conversely, iNOS levels increased by 368.96%, indicating a significant rise in oxidative stress. Treatment with RT, EXO, RT‐CPN, and the EXO + RT‐CPN mixture significantly enhanced antioxidant enzyme activity and lowered iNOS levels in the ankle compared to the arthritic group. Notably, the EXO + RT‐CPN mixture demonstrated potent effects, increasing GR, SOD, GPx, and GST expression by 404.46%, 1424.35%, 406.53%, and 294.39%, respectively, while decreasing iNOS expression by 73.58%. These results, as shown in Table 4, highlight the therapeutic potential of these treatments in mitigating oxidative damage and restoring redox balance in arthritis.

**Table 4:** Effects of RT, EXO, RT‐CPN, and EXO + RT‐CPN on antioxidant enzyme and iNOS mRNA expression in the ankle of arthritic rats.

Groups	Parameters				
	GR (fold change)	SOD (fold change)	GPX (fold change)	GST (fold change)	iNOS (fold change)
Normal control	1.078 ± 0.044^e^	1.075 ± 0.034^e^	1.040 ± 0.025^d^	1.068 ± 0.024^c^	1.079 ± 0.039^a^
Arthritic control	0.155 ± 0.051^a^	0.062 ± 0.019^a^	0.182 ± 0.056^a^	0.240 ± 0.025^a^	5.062 ± 0.453^c^
Arthritic treated with RT	0.384 ± 0.034^a,b^	0.375 ± 0.025^b^	0.372 ± 0.012^b^	0.328 ± 0.014^a^	2.341 ± 0.175^b^
Arthritic treated with EXO	0.713 ± 0.106^c,d^	0.709 ± 0.023^c^	0.650 ± 0.020^c^	0.606 ± 0.022^b^	1.618 ± 0.132^a,b^
Arthritic treated with RT‐CPN	0.488 ± 0.052^b,c^	0.4062 ± 0.016^b^	0.493 ± 0.011^b,c^	0.575 ± 0.039^b^	2.445 ± 0.067^b^
Arthritic treated with EXO + RCPN	0.781 ± 0.073^d^	0.939 ± 0.025^d^	0.923 ± 0.060^d^	0.948 ± 0.047^c^	1.338 ± 0.074^a^

*Note*: Data are expressed as mean ± standard error for groups of six rats. Identical superscript symbols in a column indicate no significant difference.

### 3.8. Effects on Various Metalloproteinases and Nrf2 Protein Expressions

**Figure 8:**
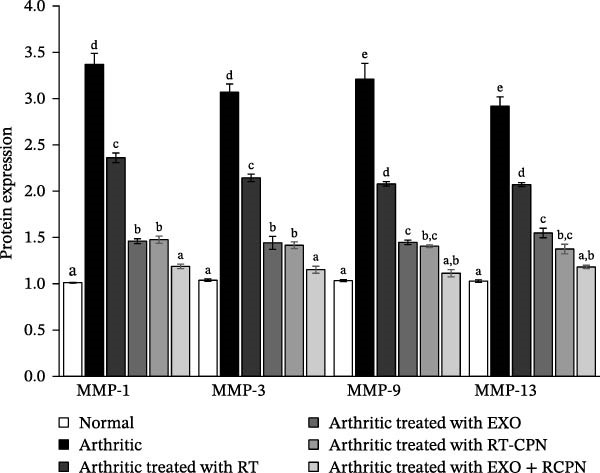
Illustrates the effects of RT, EXO, RT‐CPN, and their combination (EXO + RT‐CPN) on the protein expression of MMPs (MMP‐1, ‐3, ‐9, and ‐13) in arthritic rats. Groups sharing the same symbol (a, b, c, d and e) indicate no significant difference (number of detected samples in each group = 3).

In comparison to the control group, arthritic rats demonstrated significant upregulation of MMP‐1, MMP‐3, MMP‐9, and MMP‐13, with expression levels rising by 232.981%, 195.387%, 210.66%, and 183.785%, respectively. However, treatment with RT, EXO, RT‐CPN, and the EXO + RT‐CPN mixture effectively reduced these elevated levels. MMP‐1 expression was reduced by 29.930%, 56.680%, 56.199%, and 64.762%; MMP‐3 by 30.147%, 52.996%, 53.843%, and 62.462%; MMP‐9 by 35.257%, 54.916%, 56.184%, and 65.321%; and MMP‐13 by 29.055%, 46.956%, 52.886%, and 59.545%, respectively, compared to the untreated arthritic rats. These results show that the treatments reduce inflammation and slow the breakdown of joint tissue. In particular, the EXO + RT‐CPN combination is very effective, suggesting it could help restore joint structure and function in arthritis, as shown in Figure 8.

**Figure 9:**
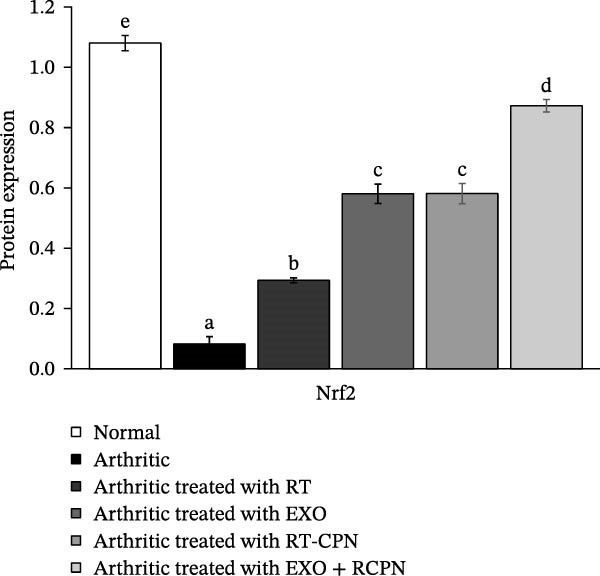
The effects of RT, EXO, RT‐CPN, and their combination (EXO + RT‐CPN) on the expression levels of Nrf2 protein in arthritic rats. Groups indicated by the same letters show no statistically significant difference (number of detected samples in each group = 3).

In arthritic rats, Nrf2 protein expression was reduced by 92.401% compared to control rats. However, treatments with RT, EXO, RT‐CPN, and EXO + RT‐CPN significantly restored Nrf2 levels, with the EXO + RT‐CPN mixture showing the strongest effect, increasing Nrf2 by 962.485%. The EXO + RT‐CPN combination is highly effective in boosting Nrf2 expression, suggesting its potential as a therapeutic strategy for arthritis by addressing oxidative stress and inflammation, as shown in Figure 9.

### 3.9. Histopathological Changes

The histological sections taken from the ankle joint of the right hind leg of normal rats demonstrated a clearly defined and intact histological structure, showing the typical synovial membrane and normal articular surfaces involving both cartilage and bone. In rats with arthritis, there were clear histological alterations, such as cartilage loss, surface irregularity, and damage to tissue. RT‐treated arthritic rats showed minor pannus development, and their articular cartilage and spongy bone were almost normal. EXO‐treated arthritic rats had considerable improvements in spongy bone and articular cartilage, almost as good as those in the normal group. The articular cartilage and spongy bone in the RT‐CPN–treated group showed healing that was nearly comparable to that of the normal group. EXO + RT‐CPN–treated arthritic rats showed almost normal articular cartilage and synovial membrane, without any signs of inflammation Figure 10.

Figure 10Photographs of hematoxylin and eosin–stained sections illustrating the impacts of RT, EXO, RT‐CPN, and EXO + RT‐CPN mixture treatments on the histological alterations in the ankle joints of arthritic rats. (A) Normal group, (B) arthritic rats, (C) arthritic rats treated with RT, (D) arthritic rats treated with EXO, (E) arthritic rats treated with RT‐CNP, and (F) arthritic rats treated with EXO + RT‐CPN combination were displayed. AC, SB, SF, BM, ER, PA, and SM are the abbreviations for articular cartilage, spongy bone, synovial fluid, bone marrow, erosion, pannus formation, and synovial membrane.

**Figure:**
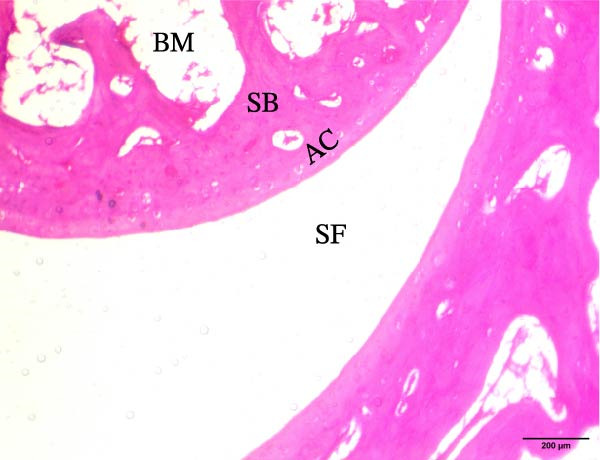
(A)

**Figure:**
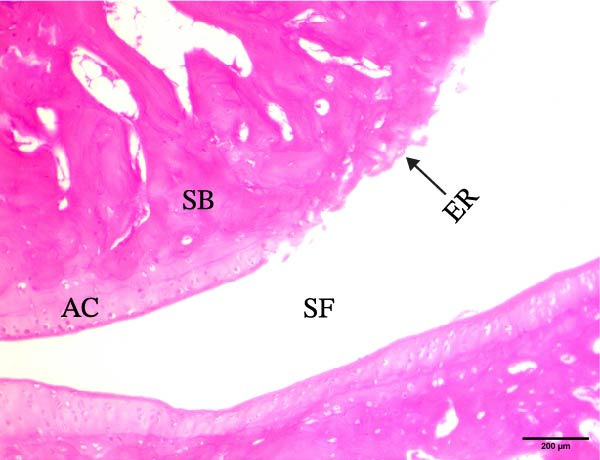
(B)

**Figure:**
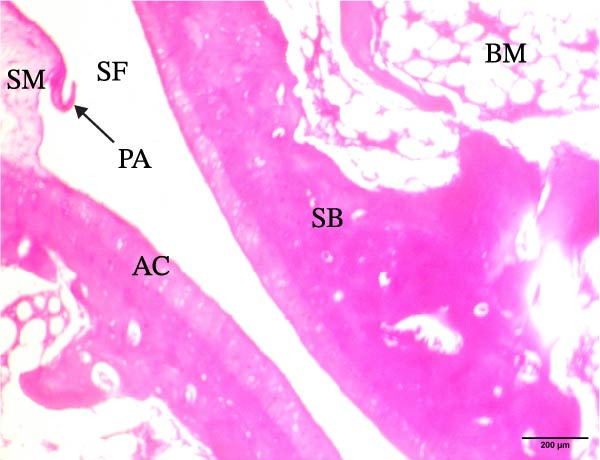
(C)

**Figure:**
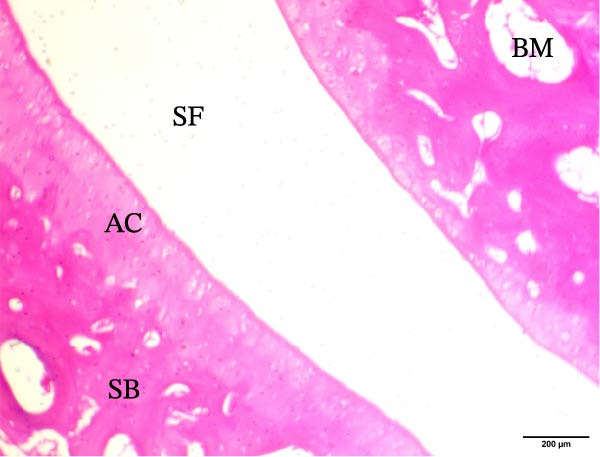
(D)

**Figure:**
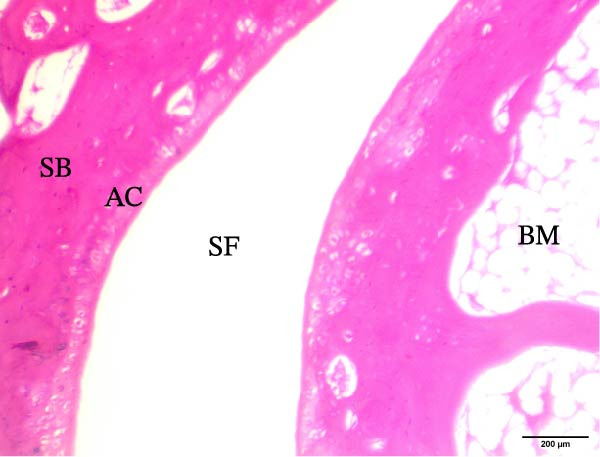
(E)

**Figure:**
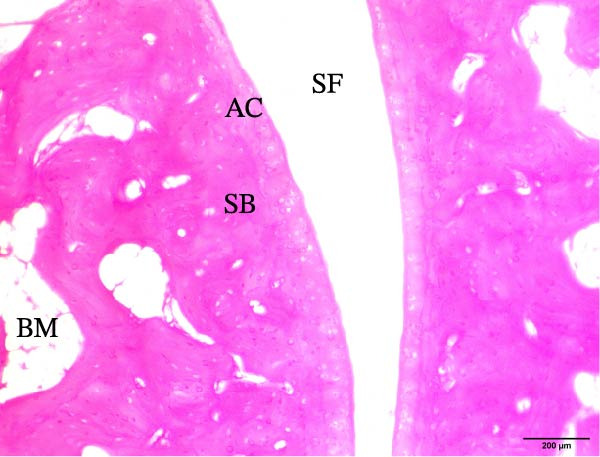
(F)

## 4. Discussion

The CFA‐induced arthritis model is widely used to evaluate the antiarthritic potential of treatments due to its similarity to human arthritis. It accurately mimics critical disease features, such as inflammatory cell infiltration in synovial tissues, cartilage degradation, and bone loss [3]. All these histopathological alterations were evidenced in histological investigations of the ankle joint of the right legs of CFA‐induced arthritic rats of the present study in association with morphological signs such as ankle and paw swelling and redness. Most researchers agree that CFA‐induced arthritis in rats is one of the most reliable models for evaluating antiarthritic drugs, as it closely mimics the pathological and inflammatory features of human arthritis [33, 34].

To assess the anti‐inflammatory properties of RT, EXO, RT‐CPN, and the EXO + RT‐CPN mixture, serum levels of ACPA, IL‐1β, IL‐6, IL‐10, and IL‐13 were measured.

**Figure 11:**
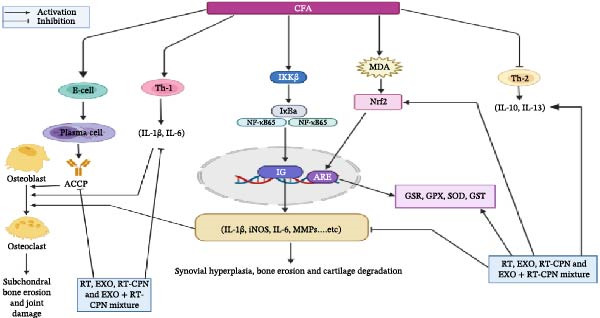
Schematic representation depicting the mechanisms of action of RT, EXO, RT‐CPN, and the EXO‐RT‐CPN combination in arthritic rats.

In arthritis pathophysiology, ACPA produced by B lymphocytes promotes joint destruction and inflammation by activating immune complexes and complement in the joints (Figure 11) [35]. ACPA particularly those directed against cyclic citrullinated peptide‐2 (CCP2), serve as a defining characteristic of RA and are regarded as reliable markers for both the onset of the disease and the severity of joint damage [36]. Like the results of Shaaban et al. [9], in our study, rats with CFA‐induced arthritis had noticeably higher serum ACPA levels. However, treatment with RT, EXO, RT‐CPN, and the combination between EXO and RT‐CPN effectively reduced this level, with the combined treatment demonstrating the strongest effect. The reduction in ACPA level suggests a downregulation of inflammatory cell activity, highlighting the potential of these treatments in mitigating arthritis‐related immune responses, which agrees with Forouzanfar et al. [10] and Ali et al. [37] that showed RT has anti‐inflammatory antioxidant. However, while EXO and RT‐CPN treatments alone showed no significant superiority over RT treatment, their combination produced remarkable improvements, nearly restoring physiological balance to that of the normal group. This highlights a strong synergistic effect between EXO and RT‐CPN, suggesting that their combined application enhances therapeutic efficacy, possibly through complementary mechanisms that amplify anti‐inflammatory, antioxidant, and regenerative responses.

Proinflammatory cytokines have been identified as key mediators in RA development through comprehensive basic and clinical research [38, 39]. These cytokines drive inflammation and contribute to bone and cartilage erosion, making them key targets for anticytokine therapies aimed at reducing disease severity and improving patient outcomes [40]. In our study, untreated arthritic rats exhibited a significant rise in serum levels of proinflammatory cytokines (IL‐1β and IL‐6) and a reduction in anti‐inflammatory cytokines (IL‐10 and IL‐13), consistent with previous findings [9, 41]. But following 28 days of RT, EXO, RT‐CPN, and EXO + RT‐CPN treatment, blood levels of IL‐1β and IL‐6 sharply declined, whereas levels of IL‐10 and IL‐13 rose, reflecting a shift toward an anti‐inflammatory profile. These results mirror earlier work that highlighted the ability of RT to mitigate inflammation [10, 12, 42] and are in line with the findings of Zhang et al. [43], who indicated that MSC‐derived EXO alleviates temporomandibular joint osteoarthritis by suppressing IL‐1β. Similarly, Arabpour et al. [44] found that MSC‐derived EXO regulates immune responses by reducing IL‐1β, IL‐6, and TNF‐α while enhancing IL‐10 and TGF‐β. The most significant therapeutic effect was observed in the EXO + RT‐CPN combination group, suggesting a potential synergistic interaction between the two treatments.

We found that rats with CFA‐induced arthritis had higher levels of MMP‐1, MMP‐3, MMP‐9, MMP‐13, and iNOS, consistent with previous findings [9, 45]. However, treatment with RT, EXO, RT‐CPN, and EXO + RT‐CPN greatly decreased the MMP protein expression and iNOS mRNA levels, with the combination therapy demonstrating the strongest effect. According to Vincenti and Brinckerhoff [46], one possible method for suppressing MMP production and delaying the onset of arthritis is direct suppression of the NF‐κB pathway in joint cells. Similarly, Pahl [47] reported that NF‐κB activation promotes inflammation by inducing iNOS expression. Although the direct involvement of NF‐κB activation was not measured in our study, our findings are consistent with previous reports suggesting that RT, EXO, and RT‐CPN may reduce inflammation by inhibiting NF‐κB activation. Based on these findings, it is possible that RT, RT‐CPN, and EXO reduce inflammation by preventing NF‐κB activation, which in turn suppresses inflammatory gene expression and reduces iNOS and MMP levels in arthritic joints. This is supported by Sun et al. [12] who reported that RT inhibits NF‐κB activation, suggesting that NF‐κB inhibition may reduce oxidative stress and proinflammatory cytokine levels in rats with CFA‐induced arthritis. Additionally, Fan et al. [48] demonstrated that bone marrow MSC‐derived EXO mitigates apoptosis and inflammation in spinal cord injury by inhibiting the TLR4/MyD88/NF‐κB signaling pathway, further highlighting their potential role in suppressing NF‐κB–mediated inflammation.

Alongside improvements in the functional and molecular markers of RA, the morphological and histological studies showed that BM‐MSC EXO and RT‐CPN treatments of arthritic rats potentially improved the histological changes of the right hind leg’s ankle joint; the combined effects were most effective in normalizing and enhancing the ankle joint’s histological integrity.

In our current study, CFA‐induced arthritic rats showed a marked rise in lipid peroxidation, indicated by elevated MDA levels, alongside a decline in antioxidant defenses, including GR, GPx, SOD, GST, and GSH (Figure 11). These findings align with those reported by Ahmed et al. [49] and Shaaban et al. [9]. However, after 28 days of treatment with RT, EXO, RT‐CPN, and the combination of EXO + RT‐CPN, we observed a remarkable upregulation in the mRNA expression of GR, GPx, SOD, and GST. Additionally, serum GSH levels increased, while MDA levels significantly declined. These effects corresponded with an increase in Nrf2 protein expression, which is essential for cellular defense against oxidative stress. In arthritic rats, the Nrf2 expression in the articular tissues of the ankle joint was markedly lower than in normal rats. Previous studies have demonstrated that Nrf2 deficiency exacerbates cartilage degradation and oxidative burden, whereas its overexpression alleviates inflammation [50]. Our findings are consistent with those of Sun et al. [12] who reported that RT treatment in a dose‐dependent manner significantly enhanced SOD, GPX, and GSH levels while reducing MDA levels in CFA‐induced arthritic rats. Similarly, Forouzanfar et al. [10] found that RT treatment significantly increased catalase and SOD activities, elevated total thiol levels, and decreased MDA levels. Moreover, Zuo et al. [51] demonstrated that BM‐MSC–derived EXO mitigate irradiation‐induced oxidative stress and promote the expression of antioxidant proteins. The observed enhancement in antioxidant enzyme levels following treatment is likely attributed to the upregulation of Nrf2 expression. Nrf2 serves as a master regulator of cellular defense mechanisms, counteracting oxidative stress by activating the transcription of antioxidant response element (ARE)–dependent genes. This regulation plays a crucial role in determining both physiological and pathological responses to oxidative insults [52–54]. In addition to its antioxidant and anti‐inflammatory functions, Nrf2 protects against mitochondrial degradation through its interaction with ARE (Figure 11) [55]. Treatment with RT, EXO, RT‐CPN, and EXO + RT‐CPN significantly enhanced antioxidant enzyme levels by specifically promoting Nrf2 expression, which in turn upregulated the gene expression of GR, GPX, SOD, and GST. These findings align with those of Ahmed et al. [42] who reported that RT increased hepatic Nrf2 expression, mediating its potent antioxidant and anti‐inflammatory effects in doxorubicin‐induced liver toxicity in Wistar rats. Furthermore, EXO plays a key role in reducing oxidative stress and inflammation in various conditions, including skin oxidation, neurological disorders, and macrophage polarization, mainly by modulating the Keap1/Nrf2 pathway [56, 57].

Among the tested treatments, the combination of EXO + RT‐CPN exhibited the most pronounced effect, synergistically enhancing Nrf2 expression and, consequently, the antioxidant response. These results illustrate the promising therapeutic effects of EXO + RT‐CPN in mitigating oxidative stress and inflammation in arthritis.

## 5. Conclusion

Based on the obtained data, it can be concluded that the treatments involving RT, RT‐CPN, EXO, and the combination of EXO + RT‐CPN have potential antiarthritic effects which may be mediated via suppressing inflammation and oxidative stress in an experimental arthritis model. Among these treatments, EXO + RT‐CPN showed the most substantial therapeutic effects, likely due to its combined ability to inhibit proinflammatory cytokines and MMPs while activating the Nrf2 pathway to strengthen antioxidant defenses. This dual mechanism not only reduces joint damage and inflammation but also promotes cellular protection and repair. These findings suggest that EXO + RT‐CPN represents a promising therapeutic strategy for arthritis, potentially offering greater benefits than individual treatments. However, further clinical studies are required to assess the safety and efficacy of the treatments, especially the combination of EXO and RT‐CPN in patients with RA.

### 5.1. Study Limitations

Further investigation, including clinical trials, is necessary to validate its safety and effectiveness in humans. The study lacks a dose‐response analysis, making the optimal doses and potential dose‐dependent effects unclear. Additionally, the observed “synergistic effect” may be additive rather than synergistic. Results from the animal model may also limit generalizability to other species or clinical settings.

**Nomenclature**
ACPAs: Anticitrullinated protein antibodies
ANOVA: One‐way analysis of variance
ARE: Antioxidant response element
CFA: Complete Freund’s adjuvant
CCP2: Cyclic citrullinated peptide‐2
CMC: Carboxymethyl cellulose
Cs: Chitosan
Cs NPs: Chitosan nanoparticles
DMARDs: Disease‐modifying antirheumatic drugs
DMEM: Dulbecco’s modified eagle medium
ELISA: Enzyme‐linked immunosorbent assay
EXO: Exosome
FCA: Freund’s complete adjuvant
GPx: Glutathione peroxidase
GR: Glutathione reductase
GSH: Glutathione
GST: Glutathione S‐transferase
IACUC: Institutional Animal Care and Use Committee
IL‐1β: Interleukin‐1β
IL‐6: Interleukin‐6
IL‐10: Interleukin‐10
IL‐13: Interleukin‐13
iNOS: Inducible nitric oxide synthase
MDA: Malondialdehyde
MMPs: Matrix metalloproteinases
MSCs: Mesenchymal stem cells
MSCs‐CM: Mesenchymal stem cell conditioned media
MVs: Microvesicles
Nrf2: Nuclear factor erythroid 2–related factor 2
NSAIDs: Nonsteroidal anti‐inflammatory drugs
PBS: Phosphate‐buffered saline
RA: Rheumatoid arthritis
RT: Rutin
RT‐CPN: Rutin‐chitosan–pectin nanocomposite
RT‐PCR: Real‐time PCR
SOD: Superoxide dismutase
TEM: Transmission electron microscopy
TNF‐α: Tumor necrosis factor
TPP: Sodium tripolyphosphate.

## Author Contributions

**Karim M. Moftah**: data curation, formal analysis, investigation, visualization, writing – original draft, writing – review and editing. **Walaa G. Hozayen**: data curation, project administration, visualization, writing – review and editing. **Nabil A. Hasona**: data curation, investigation, project administration, visualization, writing – original draft, writing – review and editing. **Hessah M. Al-Muzafar**: formal analysis, writing – review and editing, funding acquisition, project administration. **Hussah A. Alshwyeh**: project administration, formal analysis, funding acquisition, writing – review and editing. **Kamal A. Amin**: funding acquisition, project administration, writing – review and editing. **Khairy M. A. Zoheir:** investigation, visualization, writing – review and editing. **Osama M. Ahmed**: data curation, formal analysis, investigation, project administration, visualization, writing – original draft, writing – review and editing.

## Funding

The authors have nothing to report.

## Disclosure

All authors have read and approved the final version of the manuscript, agree to be accountable for all aspects of the work, and have read and agreed to the published version of the manuscript.

## Ethics Statement

The ethics committee for the care and use of animals, Faculty of Science, Beni‐Suef University, Egypt, provided suggestions, instructions, and guidelines that were followed throughout all experimentation methods (Approval Number 022/331).

## Conflicts of Interest

The authors declare no conflicts of interest.

## Data Availability

The data that support the findings of this study are available from the corresponding author upon reasonable request.
